# Hypoionic Shock Facilitates Aminoglycoside Killing of Both Nutrient Shift- and Starvation-Induced Bacterial Persister Cells by Rapidly Enhancing Aminoglycoside Uptake

**DOI:** 10.3389/fmicb.2019.02028

**Published:** 2019-09-06

**Authors:** Zhongyu Chen, Yuanyuan Gao, Boyan Lv, Fengqi Sun, Wei Yao, Yan Wang, Xinmiao Fu

**Affiliations:** ^1^Provincial University Key Laboratory of Cellular Stress Response and Metabolic Regulation, Key Laboratory of Optoelectronic Science and Technology for Medicine of Ministry of Education, College of Life Sciences, Fujian Normal University, Fuzhou, China; ^2^Engineering Research Center of Industrial Microbiology of Ministry of Education, Fujian Normal University, Fuzhou, China

**Keywords:** persister, antibiotic tolerance, aminoglycoside, antibiotic uptake, fumarate, hypoionic shock

## Abstract

Bacterial persister cells are phenotypic variants that exhibit transient antibiotic tolerance and play a leading role in chronic infections and the development of antibiotic resistance. Determining the mechanism that underlies persister formation and developing anti-persister strategies, therefore, are clinically important goals. Here, we report that many gram-negative and gram-positive bacteria become highly tolerant to typical bactericidal antibiotics when the carbon source for their antibiotic-sensitive exponential growth phase is shifted to fumarate, suggesting a role for fumarate in persister induction. Nutrient shift-induced *Escherichia coli* but not *Staphylococcus aureus* persister cells can be killed by aminoglycosides upon hypoionic shock (i.e., the absence of ions), which is achieved by suspending the persisters in aminoglycoside-containing pure water for only 1 or 2 min. Such potentiation can be abolished by inhibitors of the electron transport chain (e.g., NaN_3_) or proton motive force (e.g., CCCP). Additionally, we show that hypoionic shock facilitates the eradication of starvation-induced *E. coli* but not *S. aureus* persisters by aminoglycosides, and that such potentiation can be significantly suppressed by NaN_3_ or CCCP. Mechanistically, hypoionic shock dramatically enhances aminoglycoside uptake by both nutrient shift- and starvation-induced *E. coli* persisters, whereas CCCP can diminish this uptake. Results of our study illustrate the general role of fumarate in bacterial persistence and may open new avenues for persister eradication and aminoglycoside use.

## Introduction

Bacterial persistence is a state in which a sub-population of non-growing/slowly growing bacterial cells (i.e., persisters) resist killing by supralethal concentrations of bactericidal antibiotics (Balaban et al., 2004; Lewis, 2010). Persisters are distinct from antibiotic-resistant cells but genetically identical to their drug-susceptible kin, as their antibiotic tolerance is transient and non-inheritable (Keren et al., 2004; Lewis, 2007, 2010; Brauner et al., 2016). Because persisters have been implicated in chronic and recurrent infections (Lewis, 2010) and play a key role in the development of antibiotic resistance (Levin-Reisman et al., 2017), discovering the mechanism of persister formation and developing new strategies for persister eradication are important goals.

The formation of persisters has been attributed mainly to the entry of bacteria into a non-growing physiological state in which essential antibiotic targets are inactive and/or inaccessible to antibiotics. Genetic analyses reveal that many genes contribute to bacterial persistence (Hu and Coates, 2005; Spoering et al., 2006; Hansen et al., 2008; Lee et al., 2009; Lewis, 2010; Girgis et al., 2012; Ling et al., 2012; Shan et al., 2015; Kim et al., 2016). Well-studied components are toxin-antitoxin modules (Lewis, 2010; Germain et al., 2013; Maisonneuve et al., 2013), which produce toxins that halt cell growth and thus enable non-growing cells to tolerate antibiotics. Nevertheless, a recent study by Gerdes and colleagues raised the possibility that toxin-antitoxin modules are not involved in the formation of *Escherichia coli* persisters in unstressed conditions (Harms et al., 2017). Metabolic analyses indicate that some carbon sources are able to increase the tolerance of bacteria against one or multiple bactericidal antibiotics. For instance, Amato et al. (2013) found that diauxic shifts following exposure to fumarate or succinate can stimulate persister formation in exponential-phase *E. coli* cells (Amato and Brynildsen, 2014). Conversely, various metabolites such as glucose and mannitol may reverse the antibiotic tolerance of stationary-phase persister cells (Allison et al., 2011; Barraud et al., 2013; Meylan et al., 2017). The antibiotic tolerance of bacterial persisters appears to be tightly regulated by cellular respiration (Lobritz et al., 2015; Conlon et al., 2016; Meylan et al., 2017; Shan et al., 2017; Wang et al., 2018; Pu et al., 2019), which may affect both antibiotic uptake and downstream lethal actions of antibiotics (Lobritz et al., 2015; Meylan et al., 2017).

The use of existing antibiotics in a wiser manner, in addition to the discovery and/or development of new antibiotics, is a promising strategy for combating antibiotic-tolerant persisters (WHO, 2014; The Pew Charitable Trusts, 2016). Metabolite-stimulated aminoglycoside potentiation has been widely reported to eradicate different pathogenic persisters (Allison et al., 2011; Barraud et al., 2013; Peng et al., 2015; Meylan et al., 2017; Su et al., 2018). Iron chelators (Moreau-Marquis et al., 2009) and β-lactam aztreonam (Yu et al., 2012) were also found to potentiate the aminoglycoside tobramycin (Tom) to fight against *Pseudomonas aeruginosa* infections. Further, inhibitors of efflux pumps are potent drugs that suppress antibiotic efflux and thus increase the effective intracellular concentrations of antibiotics (Mahamoud et al., 2007; Li and Nikaido, 2009). Other promising strategies for potentiating existing antibiotics have been reported, such as pH alternation (Lebeaux et al., 2014), the use of membrane-active macromolecules (Uppu et al., 2017), and osmotic perturbation (Falghoush et al., 2017).

To study the mechanisms underlying bacterial persistence and evaluate the efficacy of antibiotics in persister eradication, a few persister models have been established and exploited. One model involves type II persisters, also called spontaneous persisters (Balaban et al., 2019), which are formed stochastically in growing cultures (Balaban et al., 2004; Maisonneuve et al., 2013; Feng et al., 2014; Brauner et al., 2016). Another is based on starvation-induced persisters (Eng et al., 1991; Keren et al., 2004; Nguyen et al., 2005), as exemplified by those formed in stationary-phase cultures and requiring a long lag time to initiate regrowth after they are transferred to growth-favorable conditions (Balaban et al., 2004; Fridman et al., 2014; Brauner et al., 2016). A third model with nutrient shift-induced persisters, which are non-growing but metabolically active cells, was proposed recently (Amato et al., 2013; Amato and Brynildsen, 2014; Kim et al., 2016; Radzikowski et al., 2016). In addition, genetically modified and environmentally stressed bacteria with high antibiotic tolerance have been explored in mechanistic studies of bacterial persistence (Xiong et al., 1996; Christensen et al., 2001; Hong et al., 2012; Wu et al., 2012; Feng et al., 2014). These enviromental factor-stimulated persisters can all be defined as triggered persisters (Balaban et al., 2019).

We recently reported that hypoionic shock (i.e., shock with an ion-free solution) can markedly potentiate aminoglycosides to kill stationary-phase *E. coli* persister cells (Jiafeng et al., 2015). We sought to expand upon our finding by examining the efficacy of this unique strategy in eradicating other persisters. Here, we report that hypoionic shock can dramatically enhance the bactericidal action of aminoglycoside antibiotics against both nutrient shift- and starvation-induced *E. coli* persisters by 2–6 orders of magnitude. This is achieved by enhancing antibiotic uptake and is apparently dependent on cellular respiration. Our work suggests potential strategies for persister eradication.

## Materials and Methods

### Strains, Medium and Reagents

Various Gram-negative (*E. coli*, *P. aeruginosa*, *Acinetobacter baumannii*, *Klebsiella Pneumoniae*, *Shigella flexneri*, *Salmonella typhimurium*, and *Aeromonas hydrophila*) and Gram-positive (*S. aureus*, *Bacillus subtilis*, *Bacillus thuringiensis*, and *Staphylococcus epidermidis*) bacterial strains were used in this study and their characteristics are described in Supplementary Table S1. For normal cell culturing, three mediums were used: M9 medium plus 5 g/L glucose, Luria-Bertani (LB) medium, or Mueller-Hinton broth. M9 medium with and without 2 g/L fumarate were used for nutrient shift- and starvation-induced *E. coli* persister formation, respectively. Yeast nitrogen broth medium was used for starvation-induced persister formation in *S. aureus*. Antibiotics used in this study include tobramycin, streptomycin, gentamicin, kanamycin, ampicillin, ofloxacin, with their manufacturers and final concentrations for different treatments being described in Supplementary Table S2. Carbonyl cyanide m-chlorophenyl hydrazone (CCCP) and its analog FCCP (carbonyl cyanide-p-trifluoromethoxyphenylhydrazone) were purchased from Sigma-Aldrich. All other chemical reagents are of analytical purity.

### Antibiotic Tolerance Test for Nutrient Shift- or Starvation-Induced Persisters

Nutrient shift-induced persisters were prepared as previously reported (Radzikowski et al., 2016). In brief, over-night cultures of each bacterial strain were diluted at 1:100 into M9 medium plus 5 g/L glucose or LB medium (37 °C, 220 rpm) and cultured to mid-exponential phase at a cell density of OD_600_ = 0.5–0.6. Cells were centrifuged and washed with M9 medium twice before transferred to M9 medium plus 2 g/L fumarate and agitated for 4 h before antibiotic tolerance test. Starvation-induced persisters were prepared as previously reported (Eng et al., 1991). Briefly, *E. coli* and *S. aureus* cells were diluted at 1:500 in Mueller-Hinton broth medium and cultured for 24 h (35 °C, 220 rpm) to a cell density of around 10^9^ CFU/mL. Cells were centrifuged, re-suspended in M9 medium (for *E. coli*) and in yeast nitrogen broth medium without amino acids (for *S. aureus*) by dilution to a cell density of around 10^8^ CFU/mL and agitated for 5 h. Antibiotic tolerance test was performed by adding each antibiotic at concentrations as described in Supplementary Table S2 and further agitated the cells for 2 or 3 h. Antibiotic-treated cells were washed twice using phosphate-buffered saline (PBS; 0.27 g/L KH_2_PO_4_, 1.42 g/L Na_2_HPO_4_, 8 g/L NaCl, 0.2 g/L KCl, pH 7.4) by centrifugation (13000 *g*, 30 s), and then 5 μL of tenfold serially diluted cell suspension were spot plated onto LB agar dishes for survival assay. The antibiotic sensitivity of each bacterium was evaluated by incubating the exponential-phase cell culture with ampicillin (100 μg/mL), tobramycin (50 μg/mL) or ofloxacin (5 μg/mL) for 2 h before bacterial survival assay.

### Aminoglycoside Potentiation by Hypoionic Shock Against Persisters

Nutrient shift- or starvation-induced persister cells were prepared as described above and hypoionic shock was performed as we previously reported (Jiafeng et al., 2015). Briefly, 100 μL cell cultures were centrifuged (13000 rpm, 1 min) in Eppendorf tube, with the supernatant being fully removed. The cells were then subjected to hypoionic shock treatment by re-suspending the pellet with pure water (i.e., without the presence of ions; a negative control was set using 0.9% NaCl solution) containing aminoglycoside antibiotic at concentrations as described in Supplementary Table S2. Cell suspension was kept at 25°C for 3 min before washing twice with PBS before subsequent cell survival assay as described above. The effect of proton motive force (PMF) and electron transport was examined by agitating the cell culture in the presence of 20 μM protonophore CCCP or FCCP, 2,4-Dinitrophenol (DNP; 20 μg/mL), rotenone (5 μg/mL) or NaN_3_ (200 μg/mL) for 1 h before hypoionic shock treatment.

### Aminoglycoside Uptake Assay

Tobramycin (gentamicin, kanamycin or streptomycin) extraction coupled with cell growth inhibition was explored for antibiotic uptake assay as follows. Briefly, 1 ml persister cells, after hypoionic shock treatment in the presence of each antibiotic at concentrations as described in Supplementary Table S2, were washed twice with PBS and re-suspended in 100 μL cell wall-digestion buffer (30 mM Tris–HCl, pH 8.0, 1 mM EDTA, 1 mg/mL lysozyme) for further incubation at room temperature for 2 h. Cells were subjected to three cycles of freezing treatment at −80°C, thermally denatured at 90°C for 10 min (Note: the bactericidal activity of each aminoglycoside after heating at 90^*o*^C for 15 min was verified to be almost fully retained; refer to Supplementary Figures S5A, S6A) and centrifuged for removing cell debris and denatured proteins. Afterward, 5 μL supernatant was spotted on *E. coli*-seeded LB agar dish for further incubation at 37°C for 8–10 h and the diameter of cell growth inhibition zone was measured. In addition, tobramycin or gentamicin uptake by CCCP or FCCP pre-treated persister cells was measured similarly. A standard curve was prepared by directly adding each aminoglycoside at different concentrations (0, 15, 25, 50, 75, and 100 μg/mL) into persister cell suspension with the cell-wall digestion buffer. The tobramycin uptake by *S. aureus* cells was measured using the same procedure except of applying a different cell wall-digestion buffer (30 mM Tris–HCl, pH 8.0) plus 20 μg/mL lysostaphin [purchased from Sangon Biotech (Shanghai) Co., Ltd.; Cat no.: A609001].

### Intracellular ATP Level Assay

A luciferase-based kit (Beyotime Biotechnology, Shanghai, China; S0026B) was used to measure ATP level according to the manufacturer’s instruction. Briefly, *E. coli* persister cells, with or without pretreatment of 20 μM CCCP for 1 h, was lysed using the lysis buffer and centrifuged (12000 *g*, 4°C, 5 min). The supernatant was quickly mixed with the working solution at equal volumes and then transferred into a 96-well plate before light recording on a FLUOstar Omega Microplate Reader using the Luminometer method.

### Proton Motive Force Assay

A flow cytometry-based assay was applied to measure the PMF by using the fluorescence probe 3,3′-Diethyloxacarbocyanine Iodide [DiOC2(3); purchased from MaoKang Biotechnology, Inc., Shanghai, China] according to the manufacturer’s instruction. Briefly, *E. coli* persisters, with or without CCCP pretreatment as described above, were diluted into PBS to a cell density of 10^6^ cells/mL and incubated with DiOC2(3) (at a final concentration of 30 μM) at room temperature for 30 min. Cells were subjected to flow cytometric analysis on FACSymphony^TM^A5 (BD Biosciences) with an excitation at 488 nm and emission at both red and green channels.

## Results

### A Shift to Fumarate as a Carbon Source for Exponential-Phase Cells Induces Persister Formation in Many Strains of Gram-Negative and Gram-Positive Bacteria

A carbon source shift from glucose to fumarate was recently reported to induce the formation of *E. coli* persister cells (Kim et al., 2016; Radzikowski et al., 2016). Here, we examined whether such a nutrient shift from glucose to fumarate could induce persister formation in other bacterial strains, including both gram-negative and gram-positive pathogens, and, if so, whether an aminoglycoside coupled with hypoionic shock could kill those persisters.

**FIGURE 1:**
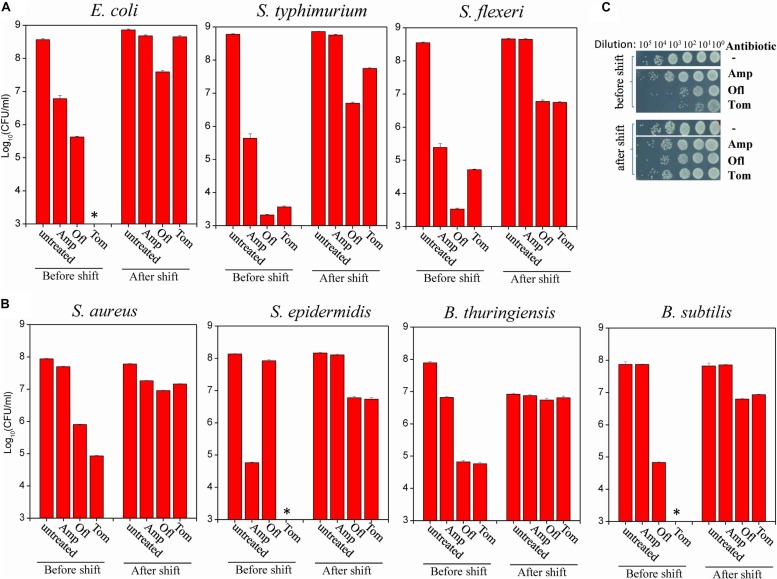
Nutrient shift to fumarate induces the formation of persisters among both gram-negative and gram-positive bacteria. **(A,B)** Survival of the indicated strains of gram-negative **(A)** and gram-positive **(B)** bacteria following a 2-h treatment with the indicated antibiotic before and after a nutrient shift to fumarate. The nutrient shift was performed by transferring exponential-phase cells (OD_600_ = 0.5–0.6) grown in LB medium to fumarate-containing M9 medium and agitating the cells for 4 h. Treated cells were spot plated on LB agar dishes to count colony-forming units. Data represent the means ± SD of three replicates; independent experiments were repeated at least three times. **(C)** Survival test of *S. aureus* cells exposed to the indicated antibiotics (Amp: 100 μg/mL; Ofl: 5 μg/mL; and Tom: 50 μg/mL) before and after the nutrient shift to fumarate. ^∗^In panels **(A,B)**: No CFU detected during cell survival assay by 100000-fold dilution.

Of the seven gram-negative bacterial strains (refer to Supplementary Table S1), we found that the tolerance of *S. typhimurium, S. flexneri*, and *E. coli* to typical bactericidal antibiotics (ampicillin [Amp], ofloxacin [Ofl], and Tom) were significantly increased by different degrees after antibiotic-sensitive exponential-phase cells grown in LB medium were transferred to fumarate-containing M9 medium 4 h prior to antibiotic treatment (Figure 1A). Meanwhile, cell densities were largely held constant before and after the nutrient shift (as indicated in the “untreated” column in Figure 1A), i.e., exponential-phase cells in a growing state were switched to a non/slowly growing state, which is a prerequisite for persister formation. *A. baumanii* Ab6 and *K. pneumonia* KP-D367 were not tested because of their antibiotic resistance (Supplementary Figures S1A,B). Both *A. hydrophila* and *P. aeruginosa* PAO1, despite tolerance to antibiotics after culture in fumarate-containing M9 medium for 4 h (Supplementary Figures S1C,D), were able to grow substantially (refer to the colony density in the red frames of figures). These strains were not used in further studies because of this growth.

Of the four gram-positive bacterial strains (refer to Supplementary Table S1), we found that the antibiotic tolerance of *S. aureus*, *S. epidermidis*, *B. thuringiensis*, and *B. Subtilis* to Amp, Ofl, and Tom were all increased after the nutrient shift to fumarate (Figure 1B; for *S. aureus*, also refer to Figure 1C). Meanwhile, their cell densities were largely held constant (as indicated in the “untreated” column in Figure 1B).

### Hypoionic Shock Facilitates Aminoglycoside Killing of Nutrient Shift-Induced *E. coli* Persisters in a Respiration-Dependent Manner

Then, prompted by our earlier observations on stationary-phase *E. coli* persisters (Jiafeng et al., 2015), we examined whether hypoionic shock could facilitate eradication of nutrient shift-induced persisters by aminoglycoside antibiotics. For this purpose, fumarate-induced *E. coli* and *S. aureus* persisters (representing gram-negative and gram-positive bacteria, respectively) and four aminoglycoside antibiotics (Tom, gentamicin [Genta], kanamycin [Kana], and streptomycin [Strep]) were tested.

**FIGURE 2:**
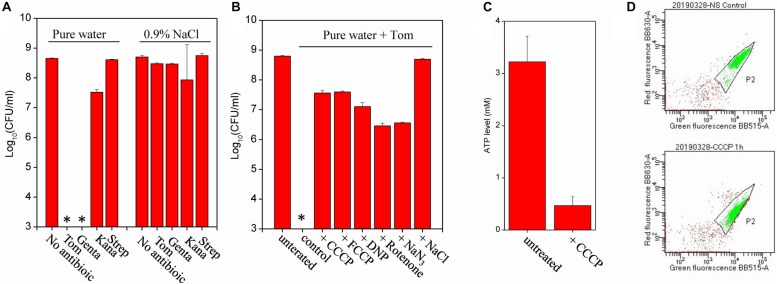
Hypoionic shock enables aminoglycoside killing of nutrient shift-induced *E. coli* persisters in a respiration-dependent manner. **(A)** Survival of nutrient shift-induced *E. coli* persisters following a 3-min treatment with the indicated aminoglycoside antibiotic dissolved in pure water (i.e., upon hypoionic shock) or in a 0.9% NaCl solution. Tom and Genta: 50 μg/mL; Kana: 100 μg/mL; and Strep: 200 μg/mL. **(B)** Survival of nutrient shift-induced *E. coli* persisters following a 3-min treatment with Tom dissolved in pure water, with persister cell pretreatment using the indicated chemicals for 1 h prior to Tom treatment. CCCP and FCCP: 20 μM; DNP: 20 μg/mL, rotenone: 5 μg/mL; and NaN_3_: 200 μg/mL. Antibiotic treatment in the presence of 0.9% NaCl was used to establish the positive control. **(C)** ATP levels in nutrient shift-induced *E. coli* persisters before and after CCCP treatment. **(D)** Results of a flow cytometric analysis of nutrient shift-induced *E. coli* persisters before (the upper part) and after (the lower part) a CCCP treatment. Cells at a density of 10^6^ cells/mL were incubated with the membrane potential fluorescence probe DiOC2(3) before analysis. Data in panels **(A–C)** represent means ± SD of three replicates; independent experiments were repeated at least three times. ^∗^In panels **(A,B)**: No CFU detected during cell survival assay by 100000-fold dilution.

Cell survival assay revealed that *E. coli* persisters could be killed by Tom- or Genta-containing pure water after the cells were resuspended in the solution and incubated for only 3 min (Figure 2A). By contrast, Tom and Genta had little effect if they were dissolved in 0.9% NaCl solution (right panel, Figure 2A), which is consistent with our early observation that the presence of ions abolished aminoglycoside potentiation (Jiafeng et al., 2015). Notably, such nutrient shift-induced *E. coli* persisters were not killed by Kana and Strep upon hypoionic shock (Figure 2A), whereas cells before the nutrient shift were killed (Supplementary Figure S2A). Time-dependent analysis revealed that the minimal time for hypoionic shock-enabled eradication of *E. coli* persisters was approximately 2 min (Supplementary Figure S2B). In contrast, fumarate-induced *S. aureus* persisters were not killed by combined treatment with aminoglycoside antibiotics and hypoionic shock (upper panel, Supplementary Figure S2C), although the same treatment enabled Tom to kill normally growing exponential-phase *S. aureus* cells [lower panel, Supplementary Figure S2C, and as reported earlier (Jiafeng et al., 2015)].

We sought to examine whether CCCP, an uncoupler of the proton gradient, could suppress hypoionic shock-potentiated aminoglycoside killing of persisters, given that the bacterial uptake of aminoglycosides requires a PMF across cytoplasmic membranes of bacteria [reviewed in Taber et al. (1987)]. For this purpose, we pretreated fumarate-induced *E. coli* persisters with CCCP for 1 h and then subjected the cells to combined treatment with Tom and hypoionic shock. Cell survival assay revealed that CCCP, as well as its functional analogs FCCP and DNP, efficiently suppressed hypoionic shock-induced Tom potentiation that could kill *E. coli* persisters (Figure 2B). We confirmed that such CCCP pretreatment decreased intracellular ATP levels (Figure 2C) and also the PMF (Figure 2D) in *E. coli* persisters, as monitored by luciferase assay and membrane potential probe-based flow cytometric analysis, respectively. In line with the results from CCCP pretreatment, rotenone and NaN_3_, two electron transport inhibitors that inhibit the transfer of electrons from iron-sulfur centers in complex I to ubiquinone and cytochrome c oxidase, respectively, were found to significantly suppress hypoionic shock-induced Tom potentiation (Figure 2B). Similarly, we found that all of these uncouplers or inhibitors significantly suppressed hypoionic shock-induced Genta potentiation that could kill nutrient shift-induced *E. coli* persisters (Supplementary Figure S2D).

### Hypoionic Shock Facilitates Aminoglycoside Killing of Starvation-Induced Persisters in a Respiration-Dependent Manner

We next examined whether hypoionic shock could facilitate aminoglycoside antibiotic killing of other persisters. Experimentally, we adopted the starvation-induced persister model described in an earlier report (Eng et al., 1991), in which stationary-phase *E. coli* and *S. aureus* cells were centrifuged and resuspended by 10-fold dilution in new medium without any nutrients, thus ruling out effects of the old medium and high cell density on antibiotic killing (Gutierrez et al., 2017).

First, we evaluated the antibiotic tolerance of starvation-adapted *E. coli* and *S. aureus* stationary-phase cells by agitating them in the presence of each aminoglycoside antibiotic for 3 h. Cell survival assay revealed that starvation adaptation caused *E. coli* to be highly tolerant to Kana and Strep and moderately tolerant to Tom and Genta (Supplementary Figure S3A) and *S. aureus* to be highly tolerant to Genta/Kana/Strep and moderately tolerant to Tom (Supplementary Figure S3B), results that are in line with those presented in an earlier report (Eng et al., 1991).

**FIGURE 3:**
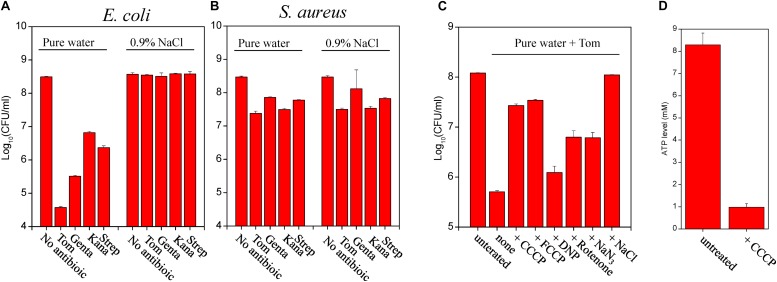
Starvation induces formation of *E. coli* stationary-phase persister cells that can be eradicated by aminoglycosides upon hypoionic shock. **(A,B)** Survival of starvation-induced *E. coli* **(A)** and *S. aureus* **(B)** persisters following a 3-min treatment with the indicated aminoglycoside antibiotics dissolved in pure water (i.e., cells in hypoionic shock) or in a 0.9% NaCl solution. *E. coli* and *S. aureus* stationary-phase cells grown in MHB medium were resuspended in M9 medium and YNB medium (without amino acids), respectively, at a final cell density of 10^8^ CFU/mL and agitated for 5 h prior to antibiotic treatment. Tom and Genta: 50 μg/mL; Kana: 100 μg/mL; and Strep: 200 μg/mL. **(C)** Survival of starvation-induced *E. coli* persisters following a 3-min treatment with Tom dissolved in pure water. Cells were pretreated using the same experimental conditions described in Figure 2B. **(D)** ATP levels in nutrient shift-induced *E. coli* persisters before and after CCCP treatment. Data in Panels **(A–D)** represent means ± SD of three replicates; independent experiments were repeated at least three times.

Next, we determined the efficacy of each aminoglycoside antibiotic coupled with hypoionic shock in killing starvation-induced persister cells. Cell survival assay revealed that starvation-induced *E. coli* persisters were killed by each aminoglycoside antibiotic upon hypoionic shock (Figure 3A), with this efficacy lost in the presence of 0.9% NaCl. Time-dependent analysis revealed that the cell survival ratio was constant with combined treatment from 1 min to 5 min (Supplementary Figure S3C), indicating that the effect of hypoionic shock on aminoglycoside antibiotics occurs as early as 1 min. Again, starvation-induced *S. aureus* persisters showed little killing after the combined treatment (Figure 3B and upper panel, Supplementary Figure S3D). However, stationary-phase *S. aureus* cells before starvation adaptation were killed by each aminoglycoside antibiotic upon hypoionic shock (lower panel, Supplementary Figure S3D).

We further examined the effect of proton gradient uncouplers (CCCP, FCCP, and DNP) and electron transport inhibitors (rotenone and NaN_3_) on hypoionic shock-induced aminoglycoside potentiation that could kill starvation-induced *E. coli* persisters. We found that both CCCP and FCCP abolished Tom potentiation by hypoionic shock and that rotenone and NaN_3_ exhibited a smaller but still significant suppressive effect (Figure 3C). We confirmed that CCCP pretreatment reduced intracellular ATP levels in *E. coli* persisters (Figure 3D) but had no significant effect on the PMF (Supplementary Figure S4), presumably because the basal PMF in starvation-induced *E. coli* persisters is quite low, as reported previously (Allison et al., 2011). Intriguingly, DNP showed a weak suppressive effect in this assay. Similarly, we found that all of these uncouplers or inhibitors significantly suppressed hypoionic shock-induced Genta potentiation for starvation-induced *E. coli* persister cell killing (Supplementary Figure S3E).

### Aminoglycoside Potentiation Upon Hypoionic Shock Is Achieved via Enhancement of Aminoglycoside Uptake by Both Nutrient Shift- and Starvation-Induced *E. coli* Persisters

In view of the fact that pretreatment with CCCP or FCCP can abolish potentiation (Figures 2B, 3C) and that aminoglycoside uptake is dependent on a PMF [reviewed in Taber et al. (1987)], we hypothesized that hypoionic shock-induced aminoglycoside potentiation is accomplished by enhancing bacterial uptake of antibiotics. Taking advantage of the high thermal stability of Tom (Supplementary Figure S5A) and the irreversible nature of aminoglycoside uptake by *E. coli* cells (Nichols and Young, 1985), we explored a protocol to measure the bacterial uptake of Tom. To this end, Tom taken up by *E. coli* cells was extracted by cell wall digestion coupled with cycled freezing/thawing and thermal denaturation, and then the relative level of Tom in the lysate was measured by bacterial cell growth inhibition assay (for details, refer to the section “Materials and Methods”).

**FIGURE 4:**
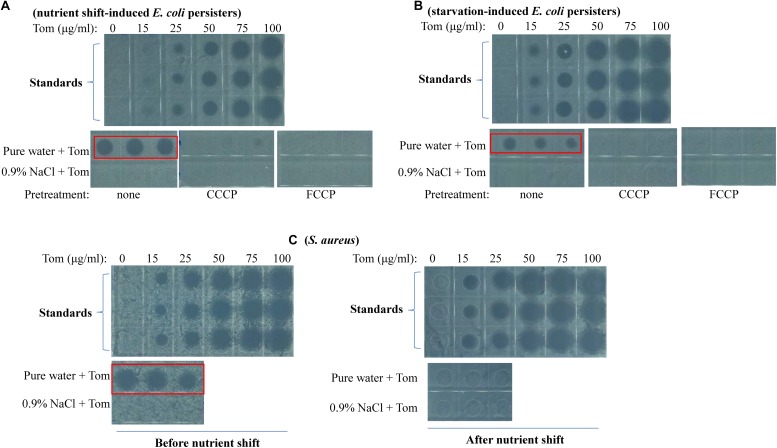
Hypoionic shock enhances the uptake of Tom by both nutrient shift- and starvation-induced *E. coli* but not *S. aureus* persisters in a PMF-dependent manner. **(A–C)** Inhibition of *E. coli* cell growth on LB agar dishes by Tom, which was extracted from nutrient shift-induced *E. coli* persisters **(A)**, starvation-induced *E. coli* persisters **(B)**, or nutrient shift-induced *S. aureus* persisters **(C)**. *E. coli* persister cells were pretreated with CCCP or FCCP for 1 h before treatment with 100 μg/mL Tom dissolved in pure water or 0.9% NaCl. Tom was extracted as described in the section “Materials and Methods.” Results of standardization were used in the quantitative analysis (refer to Supplementary Figures S5B,C). Results of Genta, Strep, and Kana uptake by *E. coli* cells are shown in Supplementary Figures S6, S7. Results of Tom uptake by stationary-phase and starvation-induced *S. aureus* cells are shown in Supplementary Figure S8. Independent experiments were repeated at least three times.

Cell growth inhibition assay revealed that Tom extracted from nutrient shift-induced *E. coli* persisters upon hypoionic shock significantly suppressed bacterial cell growth on LB agar plates (red frame, Figure 4A). In contrast, no significant cell growth inhibition was observed if Tom was extracted from the persister cells not in hypoionic shock or pretreated with CCCP or FCCP (Figure 4A). A regression analysis (Supplementary Figure S5B) based on standards (upper panel, Figure 4A) showed that the concentration of Tom extracted from nutrient shift-induced *E. coli* persister cells in hypoionic shock was up to 57 ± 4 μg/mL, whereas that extracted from the cells treated in NaCl-containing solution or pre-treated with CCCP was less than 15 μg/mL. Similarly, whereas hypoionic shock dramatically enhanced the uptake of Tom by starvation-induced *E. coli* persister cells (red frame, Figure 4B), this enhancement was diminished by the presence of 0.9% NaCl, CCCP, or FCCP pretreatment.

We also examined the bacterial uptake of Genta, Strep, and Kana during hypoionic shock. First, we verified the high thermal stability of these aminoglycosides (Supplementary Figure S6A). Then, we extracted each and measured their inhibitory effects on cell growth. Data presented in Supplementary Figure S6B revealed that Genta extracted from nutrient shift-induced *E. coli* persisters in hypoionic shock dramatically suppressed bacterial cell growth on LB agar plates (as indicated by the red frame). In contrast, the extracted Kana only slightly inhibited cell growth (Supplementary Figure S6C), and the extracted Strep hardly showed any inhibitory effect (Supplementary Figure S6D). These results agreed with the weak bactericidal actions of these antibiotics in persister cells (Figure 2A and Supplementary Figure S2A, respectively). Similarly, Genta extracted from starvation-induced *E. coli* persisters significantly suppressed cell growth (Supplementary Figure S7A), whereas extracted Kana and Strep exhibited hardly any inhibitory effects (Supplementary Figures S7B,C). These results were in accordance with the strong bactericidal action of Genta and relatively weak killing action of Kana and Strep (Figure 3A).

### Hypoionic Shock Enhances Tom Uptake by Both Exponential- and Stationary-Phase *S. aureus* Cells but Not by Nutrient Shift- and Starvation-Induced *S. aureus* Persisters

An intriguing observation in our study is that neither nutrient shift- nor starvation-induced *S. aureus* persisters were sensitive to hypoionic shock-induced Tom potentiation, whereas exponential- and stationary-phase *S. aureus* cells were sensitive (Supplementary Figures S2C, S3D). To clarify the reason for this, we measured Tom uptake by these different *S. aureus* cells. We observed that Tom extracted from exponential-phase *S. aureus* cells (i.e., cells obtained before the nutrient shift) dramatically inhibited cell growth on LB agar plates (red frame, left panel, Figure 4C), whereas Tom extracted from nutrient shift-induced *S. aureus* persister cells showed little inhibitory effect (right panel, Figure 4C). Similarly, we observed significant cell growth inhibition by Tom extracted from stationary-phase *S. aureus* cells (i.e., cells before starvation induction) (red frame, Supplementary Figure S8A), but not by Tom from starvation-induced *S. aureus* persister cells (Supplementary Figure S8B). These results suggest that the insensitivity of both types of *S. aureus* persisters to hypoionic shock-induced Tom potentiation is most likely due to the failure of hypoionic shock to enhance bacterial uptake of Tom.

## Discussion

This work resulted in several notable findings. First, we showed the general role of fumarate in inducing persisters among gram-negative and gram-positive bacteria, including those of many pathogens (Figure 1). Second, we found that hypoionic shock facilitated aminoglycoside antibiotic eradication of not only fumarate-induced (i.e., nutrient shift-induced) *E. coli* persisters but also starvation-induced *E. coli* persisters (Figures 2, 3). Importantly, we showed that hypoionic shock-induced aminoglycoside potentiation was achieved by enhancing aminoglycoside uptake and that this potentiation could be abolished by proton gradient uncouplers (Figure 4). In addition, we observed distinct activities of aminoglycoside antibiotics against cells with different growth statuses in hypoionic shock (Figures 2A, 3A and Supplementary Figures S2A,C, S3D). These findings advance our understanding of persister formation and may open avenues to the development of new anti-persister antibiotic strategies.

### Hypoionic Shock Potentiates Aminoglycosides to Kill Bacterial Persisters by Enhancing Aminoglycoside Uptake in a Respiration-Dependent Manner

We recently reported that hypoionic shock enabled aminoglycosides to kill stationary-phase *E. coli* persisters (Jiafeng et al., 2015). Here, we found that aminoglycoside antibiotics exhibited different actions against *E. coli* and *S. aureus* cells with different growth statuses upon hypoionic shock. These actions can be summarized as follows. (1) Kana and Strep eradicated exponential-phase *E. coli* cells but not nutrient-shifted *E. coli* cells (Supplementary Figure S2A), whereas Tom and Genta killed both (Figure 2A); (2) Tom killed exponential-phase *S. aureus* cells but not nutrient-shifted *S. aureus* cells, whereas the other three aminoglycoside antibiotics had little effect on either of these cell types (Supplementary Figure S2C); and (3) each aminoglycoside antibiotic eradicated stationary-phase *S. aureus* cells but not starvation-induced *S. aureus* cells (Supplementary Figure S3D). Notably, these distinct bactericidal actions of aminoglycoside antibiotics induced by hypoionic shock (Figures 2A, 3A,B and Supplementary Figures S2A,C, S3D) agreed well with the amount of aminoglycoside taken up by persister cells (Figure 4 and Supplementary Figures S6–S8), which strongly suggests that hypoionic shock-induced aminoglycoside potentiation is achieved by enhancing aminoglycoside uptake.

Hypoionic shock-induced aminoglycoside potentiation that can kill *E. coli* persisters appears to depend on the cellular respiration of bacteria based on the following evidence. First, it is well-known that aminoglycoside uptake depends on a PMF, which is generated through respiration (Taber et al., 1987). Second, recent studies have suggested that the downstream lethal action of aminoglycosides depends on the respiration of bacterial cells (Lobritz et al., 2015; Meylan et al., 2017). In our study, CCCP or FCCP alone was able to abolish hypoionic shock-induced aminoglycoside potentiation (Figures 2B, 3C and Supplementary Figures S2D, S3E) and uptake (Figure 4 and Supplementary Figures S6B, S7A). In addition, sodium azide and rotenone (inhibitors of the electron transport chain) significantly suppressed such potentiation (Figures 2B, 3C and Supplementary Figures S2D, S3E). These observations indicate that hypoionic shock, although lasting for only a couple of minutes, dramatically enhances bacterial uptake of aminoglycosides in a respiration-dependent manner.

Based on these observations, we hypothesize that certain channels on the cytoplasmic membrane of bacterial cells may be responsible for hypoionic shock-induced aminoglycoside potentiation. These channels can be activated for aminoglycoside uptake in response to hypoionic shock and may exhibit selectivity in transporting structurally different aminoglycosides, as demonstrated by the potentiation of some aminoglycosides and not others in this study. In addition, the protein level and/or transportation activity of these channels could be tightly regulated by growth conditions; therefore, they are functionally dependent on cellular respiration and physiological status. As such, an aminoglycoside (e.g., Tom) coupled with hypoionic shock can kill exponential-phase *S. aureus* cells but not these same cells after a nutrient shift to fumarate (Supplementary Figure S2C). In addition, it should be pointed out that ribosome is still the acting target of aminoglycoside during such hypoionic shock as revealed in our earlier study using the streptomycin-resistant *E. coli* strain MC4100 (Jiafeng et al., 2015).

### Clinical Potential of Hypoionic Shock-Induced Aminoglycoside Potentiation to Eradicate Persisters

Considering that the clinical application of aminoglycosides has dropped substantially in recent decades due to their toxicity and the rise of antibiotic resistance (Mingeot-Leclercq and Tulkens, 1999; Mingeot-Leclercq et al., 1999), improving the efficacy of aminoglycosides by hypoionic shock while limiting their side effects would be a clinically valuable approach. An antibiotic potentiation strategy would entail exposing subjects to aminoglycosides for only 1 or 2 min, therefore reducing the toxicity associated with aminoglycosides use. Nevertheless, this approach cannot be directly applied to curing infections in animals and humans, largely because of the ubiquity of ions throughout the animal body (e.g., Na^+^, K^+^, Cl^–^, and charged amino acids) that could abolish the potentiation effect (Jiafeng et al., 2015). If the biochemical mechanism underlying hypoionic shock-induced aminoglycoside potentiation can be discovered (e.g., if the membrane channels for aminoglycoside uptake during hypoionic shock can be identified and fully characterized), however, this would be helpful for developing new anti-persister strategies that are based on the mechanism rather than on hypoionic shock. Studies to identify this mechanism are currently underway in our laboratory.

Metabolite-stimulated aminoglycoside potentiation has recently been shown to kill stationary-phase *E. coli*, *P. aeruginosa*, and *Edwardsiella tarda* persister cells (Allison et al., 2011; Barraud et al., 2013; Peng et al., 2015; Meylan et al., 2017; Su et al., 2018). This approach has even been validated for eradication of persisters in animal models (Allison et al., 2011; Peng et al., 2015). Apparently, metabolic stimulation dramatically changes the physiological states of persister cells, boosting their respiration and reprogramming their metabolic network. It follows that persister cells might regrow during a lengthy period of metabolic stimulation (usually a couple of hours), and, if this occurs, cell regrowth would reduce the benefits of aminoglycoside potentiation to kill persisters. In comparison, our method of hypoionic shock-induced aminoglycoside potentiation requires only 1 or 2 min of stimulation [Supplementary Figures S2B, S3C; or refer to our earlier report (Jiafeng et al., 2015)]. Another advantage is that hypoionic shock enables aminoglycosides to eradicate normally growing bacterial cells [Supplementary Figures S2A,C; or refer to our earlier report (Jiafeng et al., 2015)].

### A General Role for Fumarate in Bacterial Persistence

Nutrient shift-stimulated bacterial persistence has been widely reported for *E. coli* cells that are grown in a batch culture containing two carbon sources and exhibiting diauxic growth phases (Amato et al., 2013; Amato and Brynildsen, 2014, 2015; Kotte et al., 2014). Among the carbon sources, fumarate is highly potent in increasing the formation of persisters in exponential-phase *E. coli* cells (Amato et al., 2013; Amato and Brynildsen, 2014, 2015; Kim et al., 2016; Radzikowski et al., 2016). Nevertheless, fumarate was found to impair persister formation in *P. aeruginosa* stationary-phase cells exposed to Tom by activating cellular respiration and generating a PMF through stimulation of the tricarboxylic acid (TCA) cycle (Meylan et al., 2017). These actions might be linked to the role of fumarate as a metabolite of the TCA cycle and/or as an electron acceptor (Unden et al., 2014, 2016).

Here, we have shown that a nutrient shift to fumarate is able to increase persister formation in exponential-phase gram-negative (as represented by *E. coli*, *S. typhimurium*, and *S. flexneri*) and gram-negative (as represented by *S. aureus*, *S. epidermidis*, *B. thuringiensis*, and *B. subtilis*) bacterial cells. According to the recent definition by Balaban et al. (2019), these fumarate-induced persisters should be considered type I/triggered persisters. On the other hand, the concentration of intracellular fumarate was shown to be proportional to the frequency of persisters among exponential-phase *E. coli* cells (Kim et al., 2016), illustrating its critical role in the formation of type II/spontaneous persisters [according to the definition in Balaban et al. (2019)]. Based on our findings, we propose that intracellular fumarate may converge on both external and intrinsic signals in bacterial cells and together, these signals determine persister formation, conceivably by finely tuning the electron transport chain and/or TCA cycle (Unden et al., 2014, 2016).

## Conclusion

In summary, hypoionic shock appears to facilitate aminoglycoside antibiotic killing of various types of *E. coli* persister cells, including those that are induced by nutrient shifts and starvation, as observed here, and those in the stationary phase as we previously reported (Jiafeng et al., 2015). Such aminoglycoside potentiation by hypoionic shock is most likely achieved by rapid enhancement of aminoglycoside uptake, but the precise mechanism is unknown and merits further exploration. In addition, we found that fumarate induces persisters among both gram-negative and gram-positive bacteria. Outstanding questions to be investigated include why only certain types of aminoglycoside antibiotics can be potentiated to kill *E. coli* persisters with hypoionic shock, why the sensitivity of *S. aureus* cells before and after a nutrient shift (or starvation adaption) is different in response to hypoionic shock-induced aminoglycoside potentiation, and why fumarate is able to decrease the tobramycin sensitivity of *P. aeruginosa* cells in exponential-phase growth (Supplementary Figure S1C) but increases their sensitivity in the stationary phase (Meylan et al., 2017).

## Data Availability

The raw data supporting the conclusions of this manuscript will be made available by the authors, without undue reservation, to any qualified researcher.

## Author Contributions

XF and YG designed the research. ZC, BL, FS, and WY performed the research. YW managed the project. XF, ZC, and YG analyzed the data. XF wrote the manuscript. BL, FS, and WY helped ZC to perform the research in the Figure 4C and Supplementary Figures S2D,  S3E,  S8A,B.

## Conflict of Interest Statement

The authors declare that the research was conducted in the absence of any commercial or financial relationships that could be construed as a potential conflict of interest.
