# Supplementary material for: Hypoionic Shock Facilitates Aminoglycoside Killing of Both Nutrient Shift- and Starvation-Induced Bacterial Persister Cells by Rapidly Enhancing Aminoglycoside Uptake
Source: Front Microbiol. 2019 Sep 6;10:2028. doi: 10.3389/fmicb.2019.02028 (PMC6743016; doi:10.3389/fmicb.2019.02028)
Supplement: Supplementary file 1 [file Data_Sheet_1.PDF]

Supporting information file

Hypoionic shock facilitates aminoglycoside killing of both nutrient shift- and starvation-induced bacterial persister cells by rapidly enhancing aminoglycoside uptake

Zhongyu Chen, Yuanyuan Gao, Boyan Lv, Fengqi Sun, Wei Yao, Yan Wang, and Xinmiao Fu

Table S1 Bacterial strains used in this study

**No**	**Bacterial strains**	**Origins**	**Characteristics**
1	*Escherichia coli* BW25113	Purchased from the Nara Institute of Science and Technology (Ikoma, Nara, Japan)	G^-, the parent strain (wild type) for Keio collection
2	*Pseudomonas aeruginosa* PAO1	A gift from Dr. Zhexian Tian at Peking University	G^-, kanamycin- and ampicillin-resistant
3	*Acinetobacter baumanii* Ab6	A gift from Prof. Xuanxian Peng at Sun Yat-Sen University	G^-, multi-drug resistant
4	*Klebsiella pneumoniae* KP-D367	The same as above	G^-, multi-drug resistant
5	*Salmonella typhimurium* SL1344	A gift from Prof. Xiaoyun Liu at Peking University	G^-, Streptomycin-resistant
6	*Shigella flexneri* 24T7T	The same as above	G^-, Streptomycin-resistant
7	*Aeromonas hydrophila*	A gift from Prof. Xiangmin Lin at Fujian Agriculture and Forestry University	G^-
8	*Staphylococcus aureus ATCC25923*	A gift from Prof. Luhua Lai at Peking University	G^+
9	*Staphylococcus epidermidis CMCC26069*	Purchased from Hangzhou Binhe Microorganism Reagent Co, Ltd.	G^+, Streptomycin-resistant
10	*Bacillus subtilis*	A gift from Prof. Baoyu Tian at Fujian Normal University	G^+
11	*Bacillus thuringiensis*	A gift from Prof. Zhengyu Shu at Fujian Normal University	G^+

Table S2 Antibiotics used in this study

Antibiotics	Suppliers	For antibiotic test (μg/ml)
Ampicillin (Amp)	Beijing Solarbio Science & Technology Co., Ltd.	100
Ofloxacin (Ofl)	Beijing Solarbio Science & Technology Co., Ltd.	5
Tobramycin (Tom)	Sangon Biotech (Shanghai) Co., Ltd.	50, 100 (for uptake assay)
Gentamicin (Genta)	Sangon Biotech (Shanghai) Co., Ltd.	50, 100 (for uptake assay)
Kanamycin (Kana)	Sangon Biotech (Shanghai) Co., Ltd.	100, 200 (for uptake assay)
Streptomycin (Strep)	Beijing Solarbio Science & Technology Co., Ltd	200, 300 (for uptake assay)

Figure S1

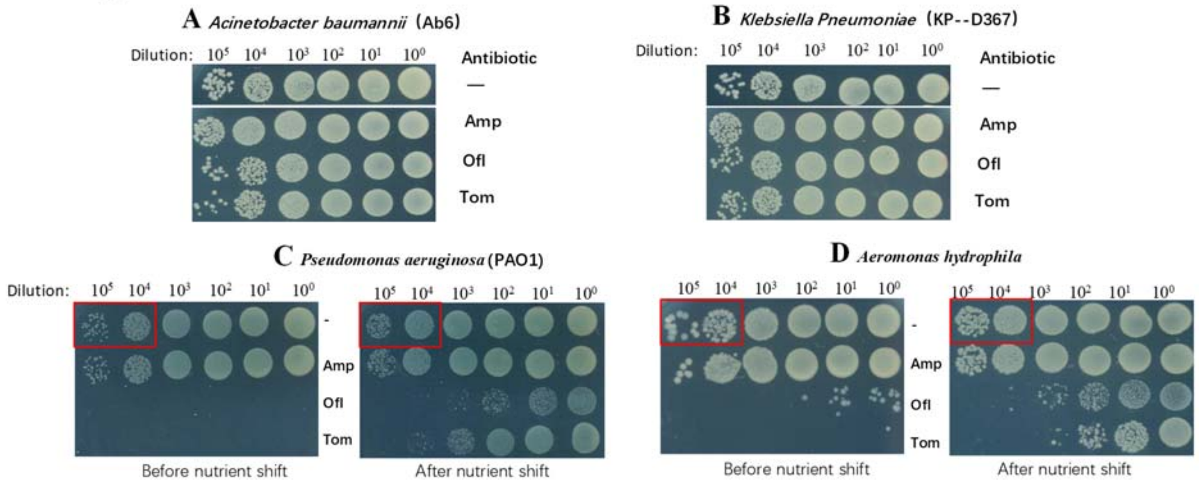

Fig. S1 Antibiotic tolerance test of several bacteria before and after nutrient shift to fumarate

(**A**, **B**) Survival of indicated gram-negative bacterial strains after exponential-phase cells (OD_{600}=0.5～0.6) grown in M9 medium plus 5 g/L glucose were agitated for two hours in the presence of indicated antibiotics. (**C** and **D**) Survival of indicated gram-negative bacterial strains after exponential-phase cells (OD_{600}=0.5～0.6) grown in LB medium were agitated for two hours in the presence of indicated antibiotics (left parts) or transferred to fumarate-containing M9 medium and agitated for four hours before the same antibiotic treatment (right parts). Note: these two bacterial strains are both able to grow in fumarate-containing M9 medium, as indicated by an apparent increase in the colony density after cultured in fumarate-containing M9 medium (refer to the colonies in the red frames).

Figure S2

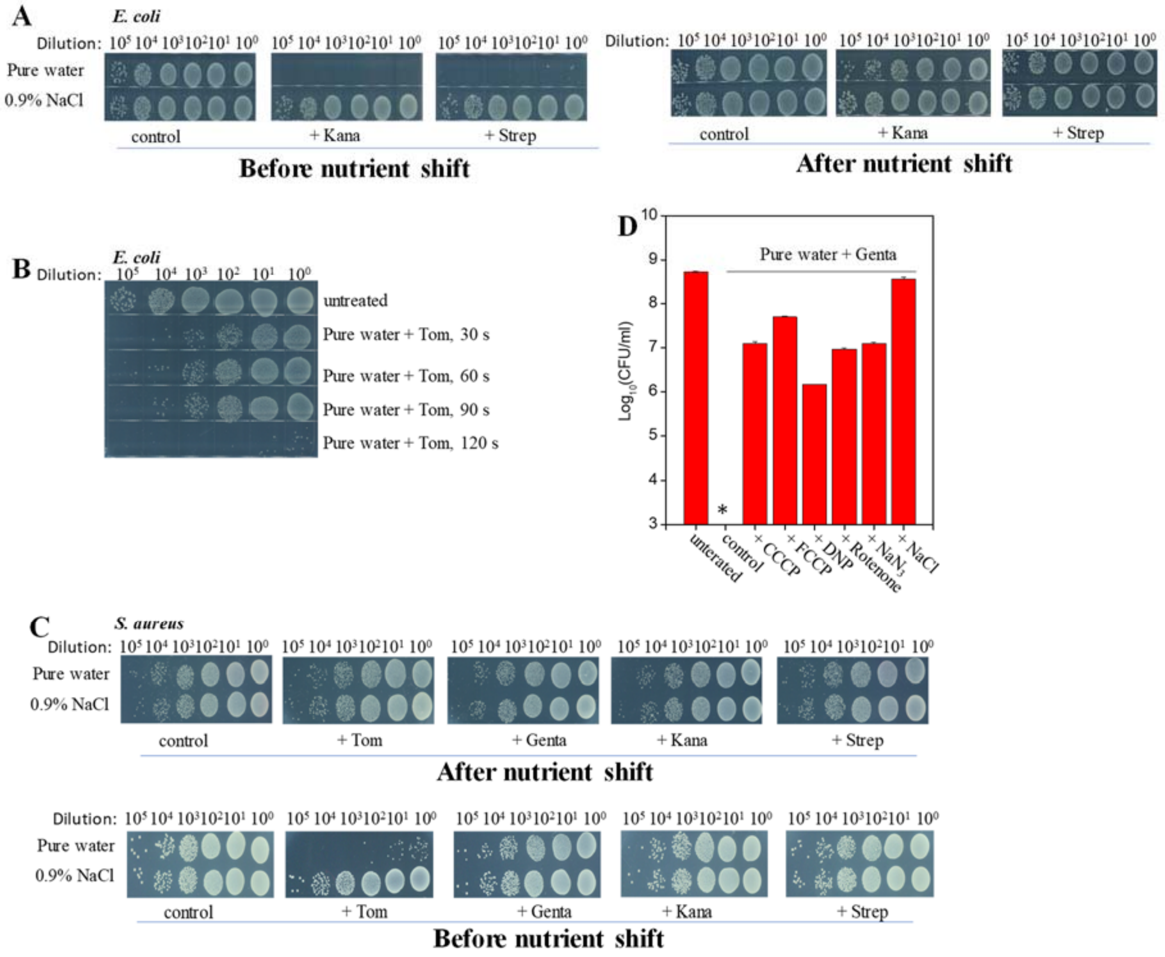

Fig. S2 Hypoionic shock facilitates aminoglycoside to kill nutrient shift-induced persister cells of *E. coli* but not *S. aureus*.

(**A**) Survival of *E. coli* exponential-phase cells following a 3-minute treatment with the indicated aminoglycoside antibiotics dissolved in pure water (i.e., cells in hypoionic shock) or in a 0.9% NaCl solution (the left part). For comparison, these cells, after nutrient shift to fumarate, were also subjected to the same treatment (the right part). (**B**) Survival of nutrient shift-induced *E. coli* persister cells following a treatment with Tom dissolved in pure water for varying length of time. (**C**) Survival of nutrient shift-induced *S. aureus* persister cells following a 3-minute treatment with the indicated aminoglycoside antibiotics dissolved in pure water in a 0.9% NaCl solution (the upper part). For comparison, *S. aureus* exponential-phase cells (i.e., cells without nutrient shift) were also analyzed (the lower part). Treated cells were washed twice with PBS and spot plated on LB agar dishes for survival assay. Tom and Genta: 50 μg/ml; Kana: 100 μg/ml; Strep: 200 μg/ml. (**D**) Survival of nutrient shift-induced *E. coli* persisters following a 3-minute treatment with Genta dissolved in pure water, with persister cell pretreatment using the indicated chemicals for one hour prior to Tom treatment. CCCP and FCCP: 20 μM; DNP: 20 μg/mL, rotenone: 5 μg/mL; NaN_3: 200 μg/mL. Antibiotic treatment in the presence of 0.9% NaCl was used to establish the positive control.

Figure S3

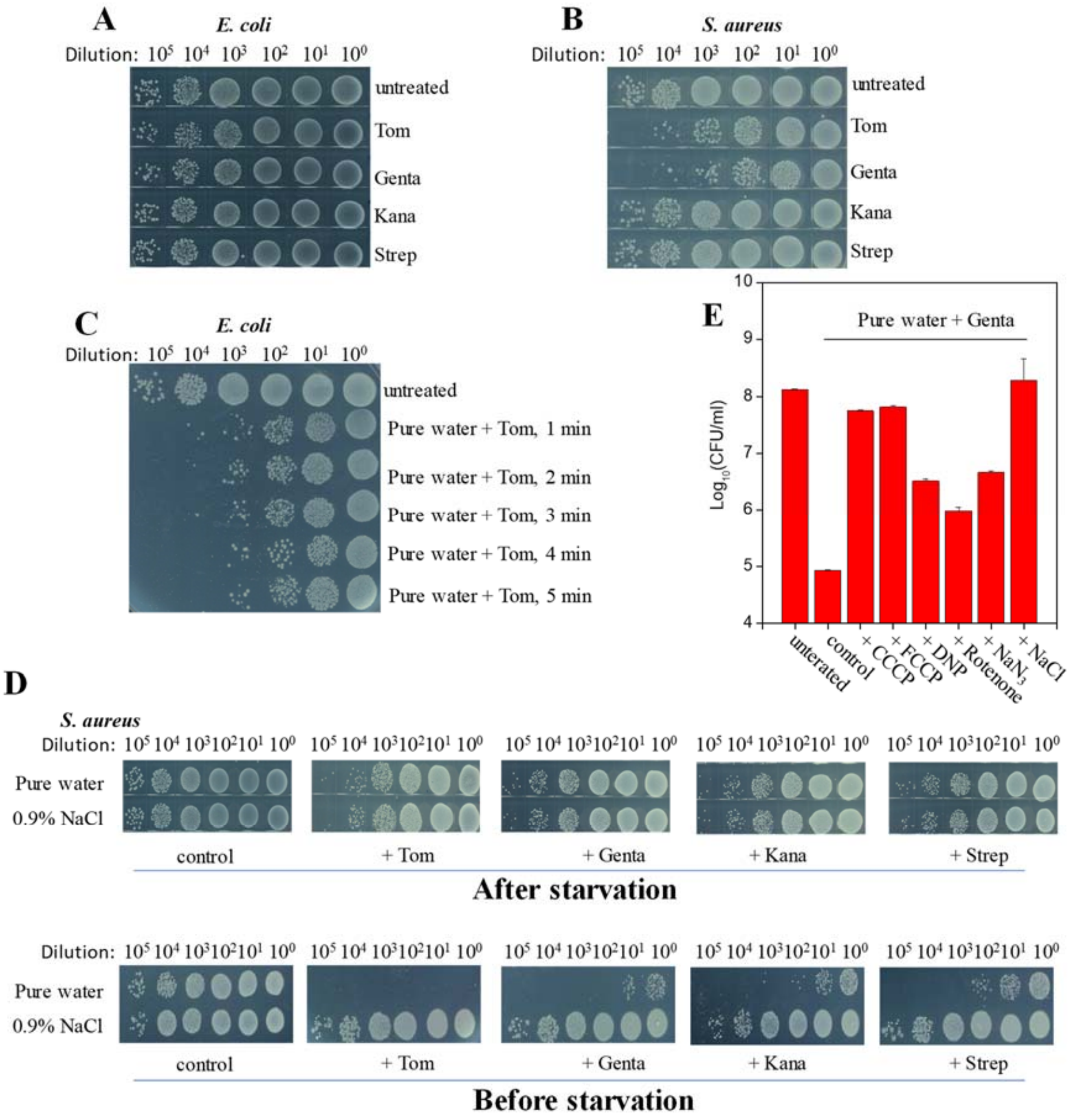

Fig. S3 Hypoionic shock facilitates aminoglycosides to kill starvation-induced persister cells of *E. coli* but not *S. aureus.*

(**A, B**) Antibiotic tolerance test of starvation-induced *E. coli* (Panel A) and *S. aureus* persisters (Panel B). *E. coli* and *S. aureus* stationary-phase cells grown in MHB medium were re-suspended by dilution into M9 medium and YNB medium (without amino acids) at a final cell density of 10^8 CFU/ml, respectively, and agitated for five hours prior to the antibiotic treatment for three hours. (**C**) Survival of nutrient shift-induced *E. coli* persister cells following the treatment with Tom as dissolved in pure water for varying length of time. (**D**) Survival of stationary-phase *S. aureus* cells following a 3-minute treatment with indicated aminoglycosides dissolved in pure water or in a 0.9% NaCl solution (the lower part). Cells were tenfold-diluted with the supernatant of the stationary-phase culture and then subjected to the treatment. For comparison, *S. aureus* stationary-phase cells after starvation induction

were also analyzed (the upper part). Treated cells were washed twice with PBS and spot plated on LB agar dishes for survival assay. Tom and Genta: 50 μg/ml; Kana: 100 μg/ml; Strep: 200 μg/ml. (**E**) Survival of starvation-induced *E. coli* persisters following a 3-minute treatment with Genta dissolved in pure water, with persister cells pretreatment using indicated chemicals for one hour prior to Genta treatment. CCCP and FCCP: 20 μM; DNP: 20 μg/ml, rotenone: 5 μg/ml; NaN_3: 200 μg/ml. Antibiotic treatment in the presence of 0.9% NaCl was set as a positive control.

Figure S4

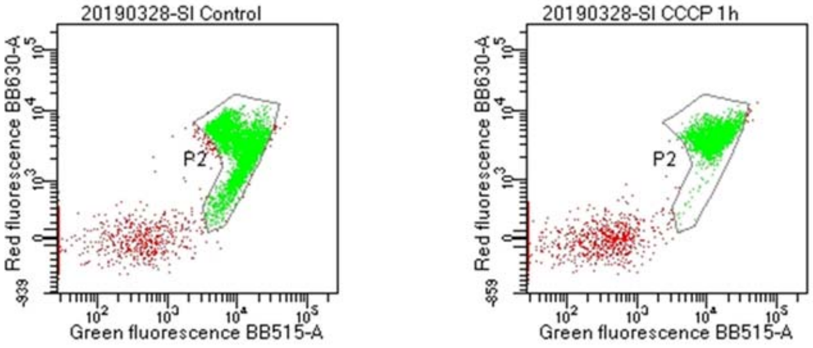

Fig. S4 CCCP pretreatment did not significantly change the PMF of starvation-induced *E. coli* persister cells.

Flow cytometric analysis results of nutrient shift-induced *E. coli* persisters before (the left part) and after one-hour CCCP treatment (the right part). Cells at a density of 10^6 cells/ml were incubated with the membrane potential fluorescence probe DiOC2(3) at a final concentration of 30 μM before analysis.

Figure S5

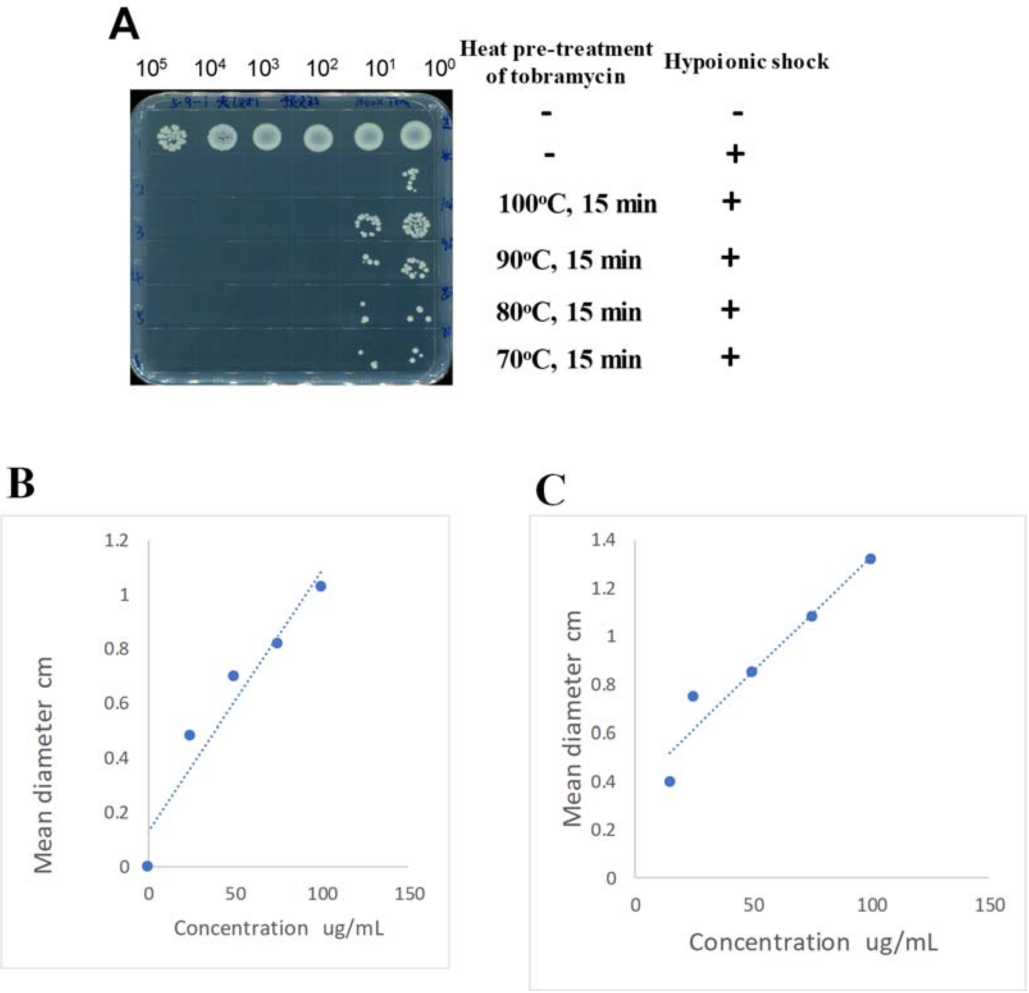

Fig. S5 Quantification of the tobramycin uptake by *E. coli* persisters

(**A**) Survival of stationary-phase *E. coli* cells following a 1-min treatment with 100 µg/ml tobramycin dissolved in pure water as we reported earlier [1], with the tobramycin-containing water being pre-heated at indicated temperatures for 15 min. This result showed the high thermal stability of tobramycin and guided us to perform tobramycin extraction experiments by thermal denaturing the cell lysates at 90°C for 15 min for removing proteins. (**B, C**) Regression analysis results for cell growth inhibition by tobramycin at standard concentration based on the results in **Figs. 4A** and **4B**. Tobramycin was directly added into the lysozyme-containing, cell wall-digestion buffer at indicated concentrations.

Figure S6

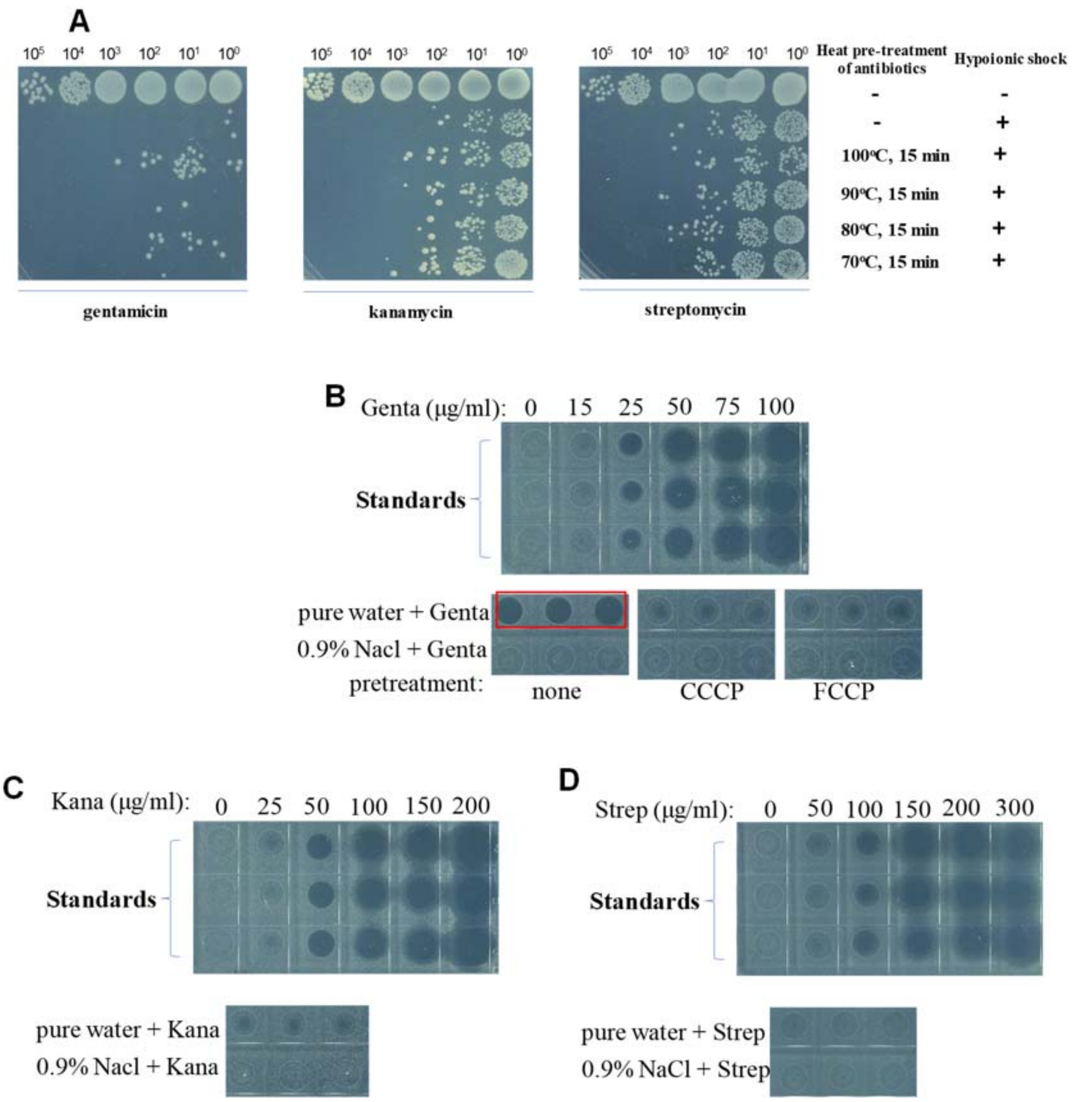

Fig. S6 Measurement of gentamicin, kanamycin and streptomycin uptake by nutrient shift-induced *E. coli* persisters

(**A**) Survival of stationary-phase *E. coli* cells following a 1-min treatment with 100 μg/ml gentamicin, 200 μg/ml kanamycin or 300 μg/ml streptomycin dissolved in pure water, with the antibiotic-containing water being pre-heated at indicated temperatures for 15 min. These results showed the high thermal stability of these aminoglycoside antibiotics and guided us to perform antibiotic extraction experiments by thermal denaturing the cell lysates at 90°C for 10 min for removing proteins. (**B, C, D**) Inhibition of *E. coli* cell growth on LB agar dishes by gentamicin (Panel B), kanamycin (Panel C) or streptomycin (Panel D), which were extracted from nutrient shift-induced *E. coli* persisters. Of these, the effects of CCCP and FCCP on the hypoionic shock-enhanced gentamicin uptake were examined (Panel B) by extracting gentamicin from CCCP- or FCCP-pretreated *E. coli* persister cells.

Figure S7

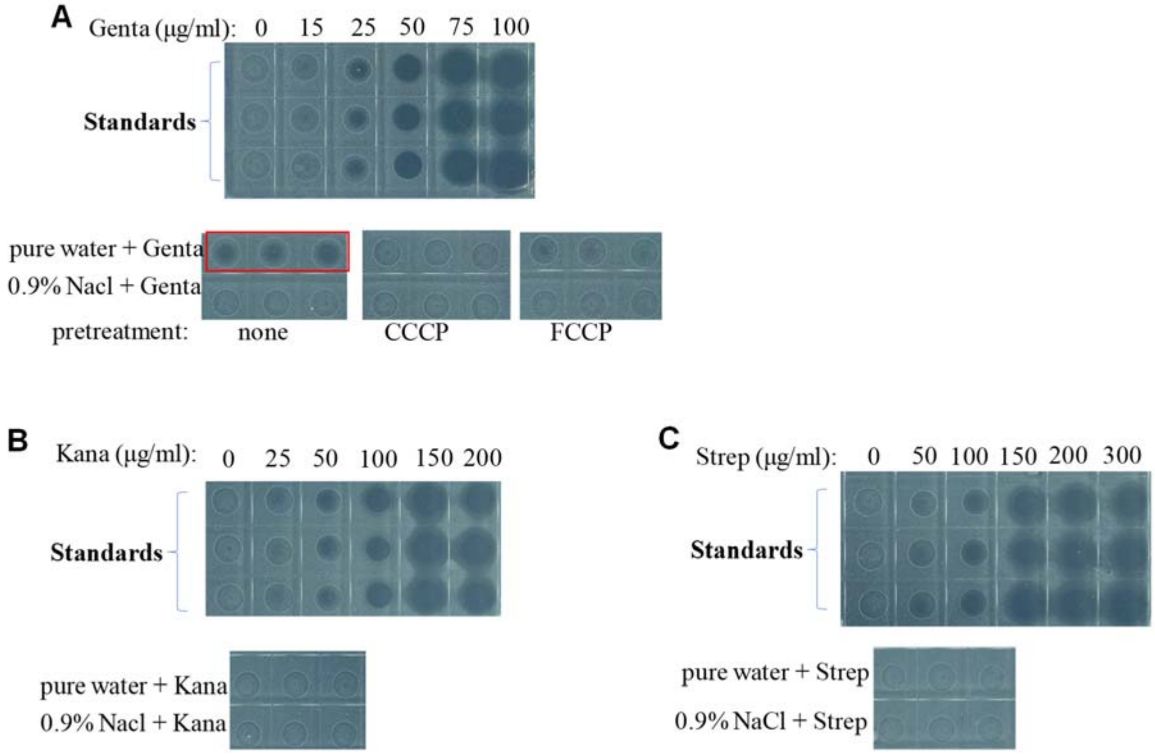

Fig. S7 Measurement of gentamicin, kanamycin and streptomycin uptake by starvation-induced *E. coli* persisters

(**A**, **B**, **C**) Inhibition of *E. coli* cell growth on LB agar dishes by gentamicin (Panel A), kanamycin (Panel B) or streptomycin (Panel C), which were extracted from starvation-induced *E. coli* persisters. Of these, the effects of CCCP and FCCP on the hypoionic shock-enhanced gentamicin uptake were examined (Panel A) by extracting gentamicin from CCCP- or FCCP-pretreated *E. coli* persister cells.

Figure S8

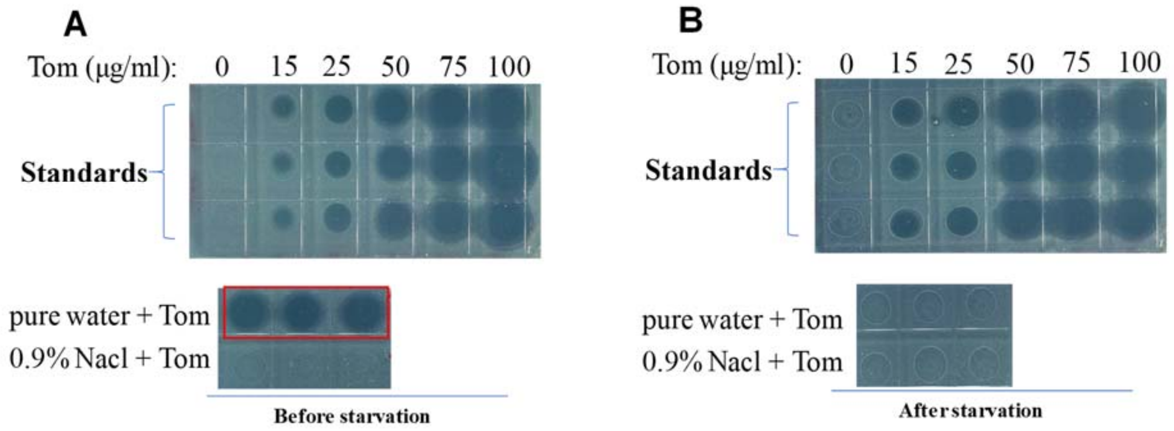

Fig. S8 The tobramycin uptake of stationary-phase *S. aureus* cells but not starvation-induced *S. aureus* persister cells is enhanced by hypoionic shock.

(**A, B**) Inhibition of *E. coli* cell growth on LB agar dishes by tobramycin, which was extracted from stationary-phase *S. aureus* cells (Panel A) or from starvation-induced *S. aureus* persister cells (Panel B).

1. Jiafeng, L., X. Fu, and Z. Chang, *Hypoionic shock treatment enables aminoglycosides antibiotics to eradicate bacterial persisters.* Sci Rep, 2015. **5**: p. 14247.
